# Evaluation and interpretation of latent class modelling strategies to characterise dietary trajectories across early life: a longitudinal study from the Southampton Women’s Survey

**DOI:** 10.1017/S000711452200263X

**Published:** 2023-06-14

**Authors:** Kathryn V. Dalrymple, Christina Vogel, Keith M. Godfrey, Janis Baird, Mark A. Hanson, Cyrus Cooper, Hazel M. Inskip, Sarah R. Crozier

**Affiliations:** 1 School of Life Course Sciences, King’s College London, London, UK; 2 MRC Lifecourse Epidemiology Centre, University of Southampton, Southampton General Hospital, Southampton, UK; 3 NIHR Southampton Biomedical Research Centre, University of Southampton and University Hospital Southampton NHS Foundation Trust, Southampton, UK; 4 NIHR Applied Research Collaboration Wessex, Southampton Science Park, Innovation Centre, 2 Venture Road, Chilworth, Southampton, SO16 7NP, UK; 5 Institute of Developmental Sciences, Faculty of Medicine, University of Southampton, Southampton, UK; 6 NIHR Oxford Biomedical Research Centre, University of Oxford, Oxford, UK

**Keywords:** Trajectory modelling, Growth mixture models, Group-based trajectory modelling, Lifecourse epidemiology, Diet quality

## Abstract

There is increasing interest in modelling longitudinal dietary data and classifying individuals into subgroups (latent classes) who follow similar trajectories over time. These trajectories could identify population groups and time points amenable to dietary interventions. This paper aimed to provide a comparison and overview of two latent class methods: group-based trajectory modelling (GBTM) and growth mixture modelling (GMM). Data from 2963 mother–child dyads from the longitudinal Southampton Women’s Survey were analysed. Continuous diet quality indices (DQI) were derived using principal component analysis from interviewer-administered FFQ collected in mothers pre-pregnancy, at 11- and 34-week gestation, and in offspring at 6 and 12 months and 3, 6–7 and 8–9 years. A forward modelling approach from 1 to 6 classes was used to identify the optimal number of DQI latent classes. Models were assessed using the Akaike and Bayesian information criteria, probability of class assignment, ratio of the odds of correct classification, group membership and entropy. Both methods suggested that five classes were optimal, with a strong correlation (Spearman’s = 0·98) between class assignment for the two methods. The dietary trajectories were categorised as stable with horizontal lines and were defined as poor (GMM = 4 % and GBTM = 5 %), poor-medium (23 %, 23 %), medium (39 %, 39 %), medium-better (27 %, 28 %) and best (7 %, 6 %). Both GBTM and GMM are suitable for identifying dietary trajectories. GBTM is recommended as it is computationally less intensive, but results could be confirmed using GMM. The stability of the diet quality trajectories from pre-pregnancy underlines the importance of promotion of dietary improvements from preconception onwards.

Poor diet quality is arguably one of the leading determinants of non-communicable diseases, including obesity, CVD and some cancers^(1)^. A recent Global Burden of Disease study showed that poor diet quality is associated with more than 20 % of deaths. Half of these were attributed to unfavourable levels of intake of wholegrains, fruits and Na^(2)^. In the UK, the latest findings from the National Diet and Nutrition Survey suggested that adults consume too much salt, saturated fats and free sugars, and only 33 % consume the recommended five daily portions of fruit and vegetables^(3)^. For children, although consumption of free sugars has decreased in recent years, fibre, saturated fats and fruit and vegetable intakes are not optimal. During the early years, nutritional intake is influenced by maternal preconception^(4)^ and antenatal diet^(5)^, as well as environmental, lifestyle and genetic factors^(6)^. To improve population health, it is imperative that we identify patterns in diet quality and time points within the lifecourse most likely to benefit from nutritional interventions.

In nutritional sciences, a common approach for exploring relationships between diet quality, health and disease focuses on nutrient intakes assessed at one time point, and their subsequent associations with health outcomes^(7,8)^. However, diet is a complex exposure variable as it can be difficult to measure accurately. More recently, principal component analysis (PCA) has been applied as an alternative approach to reveal dietary patterns and explore their associations with long-term health outcomes^(9,10)^. PCA reduces dietary data into fewer variables and conceptually illustrates a broader picture of an individual’s habitual diet, and so it may provide a stronger explanation of the relationship between diet and disease risk than individual nutrients or foods^(11)^. Furthermore, due to the increasing availability of repeated observations from population cohorts, longitudinal analyses of dietary data are becoming more common^(12,13)^. These studies have explored relationships between an average trajectory over time of a specific nutrient or eating behaviour and outcomes of interest. These analyses were limited to two or three waves of data, and a population average is unable to identify subgroups within a given dataset. An alternative method for modelling longitudinal data, which has frequently been applied to growth data^(14,15)^ and more recently to eating behaviour^(16)^ and lifestyle patterns in childhood^(17)^, is classifying individuals into subgroups using latent class methodologies. The objective of these approaches is to model information about inter-individual differences in intra-individual change over time^(18)^. These methods can be applied to model dietary pattern trajectories and may be able to identify time points across the lifecourse or population groups at risk of poor diet quality.

The aim of this study was to evaluate methods for trajectory modelling of diet quality indices (DQI). The first two objectives were to provide a practical overview of (1) group-based trajectory modelling (GBTM), a form of latent class growth analysis, and (2) growth mixture modelling (GMM), applied to a DQI obtained from women and their offspring from the UK Southampton Women’s Survey (SWS). The SWS collected data from young non-pregnant women and followed up those who became pregnant and their offspring up to 8–9 years of age and beyond. It is the only population cohort in Europe with data including dietary behaviours collected prospectively from before the women became pregnant. We have provided a summary of the two latent class modelling strategies, including evaluation and interpretation of model adequacy assessment as well as strengths and weaknesses. Furthermore, to assess the similarity between methods, we have used cross-tabulations and correlation coefficients. For the third objective, we compared these methods with an approach we have previously used to describe dietary trajectories in the SWS cohort that converted the continuous DQI into thirds at each assessment point^(19)^. This paper focuses on the application of these methods. We have discussed the relationship between early-life dietary trajectories and childhood health outcomes elsewhere^(20)^.

## Methods

### Southampton Women’s Survey

#### Population

The SWS is a cohort of women and their children born in the city of Southampton, UK. Full details of the study have been published^(21)^. In brief, from April 1998 to December 2002, 12 583 initially non-pregnant women aged between 20 and 34 years were recruited and pre-pregnant characteristics obtained (education, social class, lifestyle, diet and anthropometry). Subsequently, 3158 became pregnant and delivered a live-born singleton infant; these women were invited to attend face-to-face follow-up appointments during their pregnancy (11-, 19- and 34-week gestation). The offspring were studied at birth, and follow-ups performed across infancy (6 and 12 months) and childhood (2, 3, 4, 6–7 and 8–9 years). All interviews with participants were performed by trained research nurses.

#### Ethics

The SWS was conducted according to the guidelines laid down in the Declaration of Helsinki and was approved by the Southampton and South West Hampshire Local Research Ethics Committee (08/H0502/95). Written informed consent was obtained from all participating women and by a parent or guardian with parental responsibility on behalf of their children.

### Diet quality index

#### FFQ

In the mother–child dyads, diet was assessed at eight time points. Maternal dietary data were recorded at the preconception, and 11- and 34-week gestation visits^(22)^. Mothers’ food intake over the previous 3 months was assessed using a 100-item validated FFQ^(22)^. For the offspring, questionnaires were administered by trained research nurses to the child’s parent or guardian. Dietary intake was assessed using age-specific FFQ when they were aged 6 and 12 months and 3, 6–7 and 8–9 years of age^(23–25)^. At the age of 6 months, food intake was assessed over the previous 7 d using a thirty-four-item FFQ^(23)^. At 12 months, food intake was assessed over the previous 4 weeks using a seventy-eight-item FFQ^(24)^. At ages 3, 6–7 and 8–9 years, food intake was evaluated over the preceding 3 months. At the 3 and 6–7 year visits, diet was assessed using an eighty-item FFQ^(25)^. At the 8–9 years’ visit, a thirty-three-question FFQ derived from the eighty-item FFQ was administered due to participant time restrictions; the questions selected were based on evidence of an association between specific food groups and adiposity^(26)^ and foods found to be discriminatory on a dietary quality score^(27)^.

#### Principal component analysis

At each time point, the foods listed in the corresponding FFQ were categorised into groups based on similar nutritional composition (e.g. carrots, parsnips, swedes and turnips were included in the ‘root vegetables’ group; bacon, ham, corned beef, meat pies and sausages were included in the ‘processed meats’ group), and PCA was performed on the reported weekly frequencies of consumption of the food groups. For each time point, the first principal component was found to describe a ‘diet quality index’ (DQI); a high score was associated with frequent consumption of healthy foods recommended in government guidelines and less frequent consumption of less healthy foods that contribute to diet-related disease. In previous SWS publications, the DQI has been referred to as an infant guidelines score^(28)^ at 6 and 12 months of age, as well as a prudent diet score^(29)^ in the mother and in the children at ages 3 and 6–7 years. Participants with a high score for these types of dietary patterns conformed with dietary recommendations, while those with a low score did not follow them. At each assessment, the DQI were transformed (Fisher–Yates) to a mean of 1 and a sd of 1^(10)^. Full details of these analyses, including validation of the FFQ, have been published^(19,23–25,30)^.

#### Latent class trajectory strategy

For the latent class trajectory modelling, we used the repeatedly measured DQI (continuous variable) collected at eight time points from preconception to 8–9 years of age. GBTM and GMM were selected over other latent class modelling strategies because they are able to handle missing data (under the missing at random assumption) and unevenly spaced assessments over time *(*e.g. *6 months, 12 months and 3 years of age)* ^(31,32)^. We applied the following steps to GBTM and GMM to identify the appropriate number of latent classes for the DQI trajectories. All analyses were performed in Stata 15.0.

### Step 1: Modelling longitudinal data

#### Part 1a

Before starting latent class modelling, it is beneficial to model the individual diet quality trajectories for all participants using a spaghetti plot. This may identify patterns or subgroups within a given dataset and help estimate the appropriate number of latent classes.

#### Part 1b

The second part is to fit a growth curve model (single trajectory). These are also described in the literature as latent trajectory models or latent growth curve models^(33)^. Rather than categorising individuals into subgroups, this approach delineates the strength, direction and average pattern for the entire sample^(34)^. This model is fitted in Stata using the *xtmixed* command. The DQI is the dependent variable and time as the independent variable (fixed part of the model). Participant ID is included in the random part of the model. The results for this output have random intercepts and coefficients for each time point and estimate the mean change in diet quality over time.

### Step 2: Model specification

To identify the optimal number of latent classes for the DQI, we used a forward modelling approach from one to six classes as advised by the GRoLTS checklist Guidelines for Reporting on Latent Trajectory Studies^(33)^. After fitting the one-class model, we incrementally added extra classes and investigated the model adequacy assessments discussed below. Once the model adequacy stopped improving, we fitted an additional model with one extra class to ensure the full array of possible models had been tested.

### Step 3: Model estimation

Each model was assessed using the following criteria: the Akaike information criterion (AIC); the Bayesian information criterion (BIC); average posterior probability of assignments; the ratio of the odds of a correct classification; group membership and relative entropy.

#### Likelihood-based statistics

AIC and BIC are likelihood-based statistics; BIC favours more parsimonious models compared with the AIC^(35)^. For both statistics, a value closer to 0 implies better model fit^(36)^.

#### Classification statistics

For each participant in a model, the average posterior probability of assignment was calculated. This value represents the average posterior probability of belonging to a class over all the individuals assigned to a class. A class average of the average posterior probability of assignment should be above 70 %, which indicates that the individuals assigned to a trajectory follow a similar pattern over time^(37)^. The odds of a correct classification is the ratio of the odds of a correct classification into each group on the basis of the maximum probability classification rule and the estimated class membership. Each class should hold a group membership of at least 5 %. However, this is dependent on sample size. The minimum sample size recommended for latent class modelling is between 300 and 500^(35)^, but if there is a much larger sample size then group membership can be less than 5 %. Relative entropy estimates the accuracy (convergence) of classification of individuals into the different latent classes. Entropy values close to 1 indicate lower classification uncertainty.

### Step 4: Model selection and interpretation

To determine the optimum number of latent classes there are several factors to consider, including the research question, parsimony, the assessment criteria and interpretability. The BIC value is commonly used to assess the appropriate number of latent classes. However, BIC values may decrease as more classes are added reflecting model overfit^(36)^ and, therefore, this value might not always provide the optimum selection criteria. For our analysis, the number of classes chosen was based on the lowest BIC and satisfactory values for the remaining criteria. We also compared the findings between GBTM and GMM to confirm the correct number of latent classes.

### Latent class methods

#### Group-based trajectory modelling (Stata traj command)^(38)^

GBTM is a semi-parametric technique used to identify distinct trajectories^(31)^. Although each individual in the SWS data has a distinct diet quality pattern and distinct changes in their pattern over time, GBTM allows for the distribution of individual differences within the data to be clustered. Given that the strength and direction of change can vary for each trajectory, an intercept and slope are generated for each trajectory. GBTM fixes the slope and the intercept equally across individuals within a trajectory (class). Additionally, GBTM can handle trajectories in the same model that follow a different pattern/shape (e.g. intercept, linear, quadratic and cubic)^(29)^. At least three waves of data are needed to accommodate a quadratic shape. If there are four or more data points, this can accommodate a cubic shape. With reference to the SWS data, we cannot assume that all participants in a given sample would experience the same longitudinal changes in diet quality, especially during pregnancy or in the early years when diet quality may be affected by food aversion or neophobia^(39)^. Therefore, the application of the same shape for all trajectories could hide these group differences. When applying GBTM, the intercept, linear, quadratic and cubic functions of each trajectory were tested. To ensure model parsimony, non-significant cubic and quadratic terms were removed from trajectories. However, linear parameters were retained irrespective of significance as long as the BIC was lower than if an intercept parameter was used^(40)^. This process was repeated until there was no evidence of an improvement in model fit assessed by BIC.

#### Growth mixture model (Stata gllamm command)^(41)^

GMM is a parametric technique. Unlike GBTM, it is a form of latent class growth analyses that allows for random effects^(42,43)^. GMM estimate a mean growth curve for each class or trajectory and use random effects to summarise individual differences within a class. This heterogeneity within classes is captured by the intercept and slope for each class^(44)^. Therefore, these random effects are used to represent the gap between individuals’ latent growth parameters and the population’s mean growth parameter. Unlike the ‘traj’ command for GBTM, which requires the data to be in wide format, GMM with the ‘gllamm’ command requires the data to be in long format.

### Cumulative effect of diet quality

In a previous SWS analysis, we derived diet quality trajectories across childhood by converting the continuous DQI into thirds at each assessment point^(19)^. To compare this approach with the GBTM and GMM, at each time point participants were assigned a value of 0 (lowest), 1 (middle) or 2 (highest) according to where their diet quality score lay in the distribution. These values were summed to obtain a DQI across early life, ranging from 0 (lowest diet quality) to 16 (highest diet quality). If a participant was missing an assessment point, the average value from their assessments was substituted for the missing value. The DQI across early life was used as a categorical variable (grouped as 0, 1–4, 5–9 and 10–15, 16).

### Statistical analysis for demographic characteristics

For demographic statistics, binary and categorical variables are presented using counts and percentages. The distributions of continuous variables were assessed using coefficients of skewness and then summarised by mean and standard deviation for normally distributed variables, or median and interquartile range for non-normally distributed variables.

## Results

### Southampton Women’s Survey

**Fig. 1.**
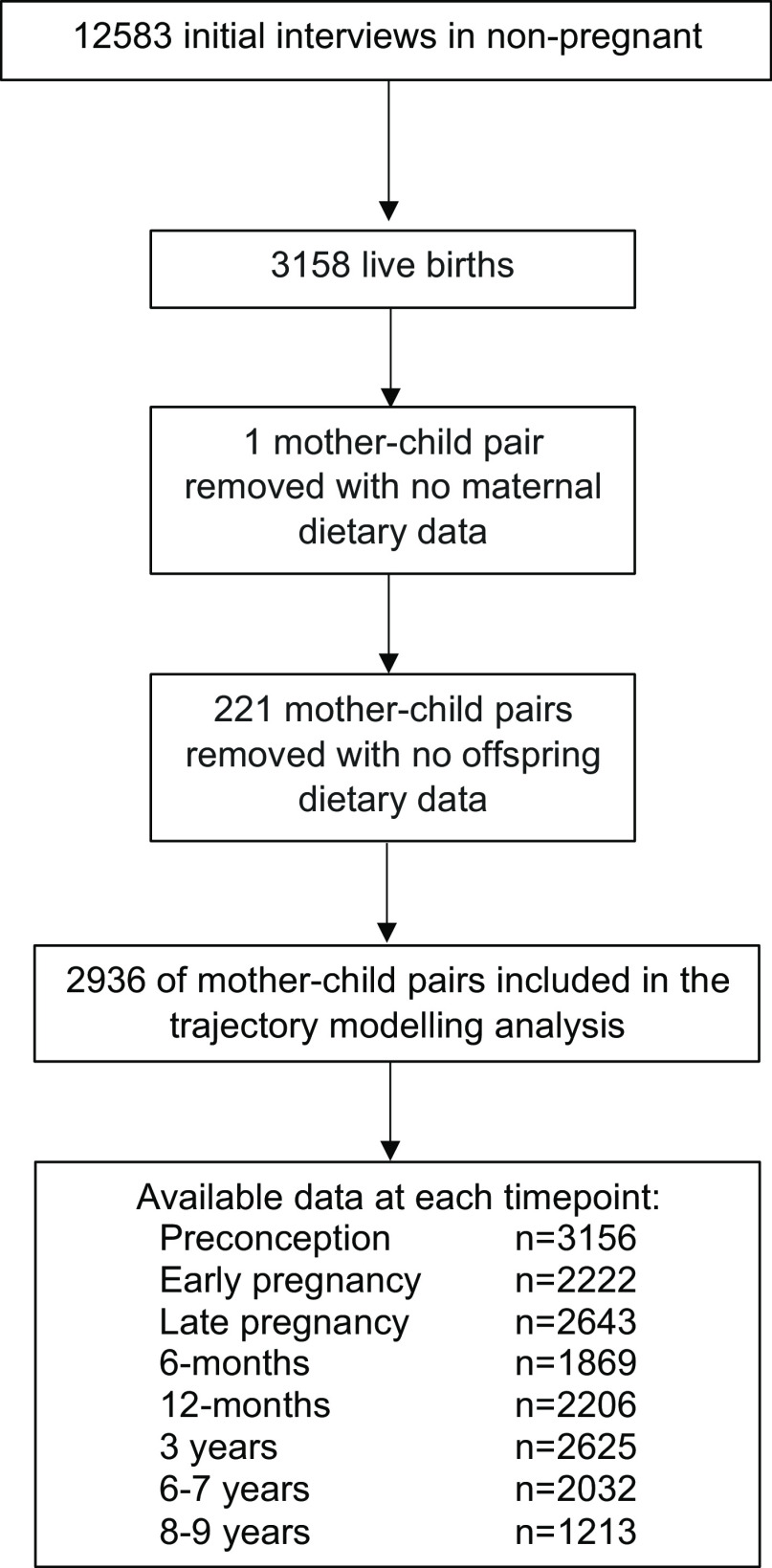
Flow diagram.

Of the 3158 mothers who gave birth to live-born infants in the SWS, we excluded mother–child dyads if the mother (*n* 1) or the child (*n* 221) were missing all of their dietary assessment points (Fig. 1). Therefore, 2936 SWS women and their children were included in the final analysis. Table 1 details demographic characteristics for the cohort. For the mothers, the median BMI at the preconception visit was 24·1 kg/m^2^ (interquartile range 21·8–27·3), and 3 % had no formal educational qualifications. Ninety-nine per cent were White, 15 % smoked in pregnancy and 48 % were multiparous at study recruitment. Mean maternal age at birth was 30·7 years. Eighteen per cent of mothers did not breastfeed, 41 % breastfed for < 3 months, 32 % breastfed for 4–11 months and 9 % continued breast-feeding for > 12 months.

**Table 1.** Demographic characteristics of 2936 mother–child pairs from the Southampton Women’s Survey

		*n* Mean (sd)/*n* (%)/Median (IQR) ^a^		
Maternal			*n*	%
BMI (kg/m^2^)				
Median		2910	24·1	
IQR			21·8–27·3	
Highest qualification level	None		85	3
	CSE		264	9
	O levels		838	29
	A levels		897	31
	HND		190	6
	Degree		654	22
Ethnicity (White)		2936	2812	96
Ever smoked in pregnancy		2802	432	15
Parity (multiparous)		2934	1418	48
Age at birth (years)				
Mean		2936	30·7	
sd			3·8	
Family				
Dominant social class	Professional		295	10
	Management/technical		1227	42
	Skilled (non-manual)		860	30
	Skilled (manual)		287	10
	Partly skilled		199	7
	Unskilled		23	1
Child				
Breast-feeding	Never tried		503	18
(months)	< 1		570	20
	1–3		598	21
	4–6		478	17
	7–11		417	15
	12 or more		240	9
Gestational age at delivery (weeks)				
Mean		2936	39·8	
sd			1·8	
Birth weight (g)				
Mean		2909	3442	
sd			548·0	
Sex (female)		2936	1405	48

IQR, interquartile range; *n*, number; CSE, Certificate of Secondary Education; HND, Higher National Diploma.

^a^Binary and categorical variables are presented using counts and percentages. The distribution of continuous variables was assessed using coefficients of skewness and then summarised by mean and standard deviation or median and IQR where appropriate.

**Fig. 2.**
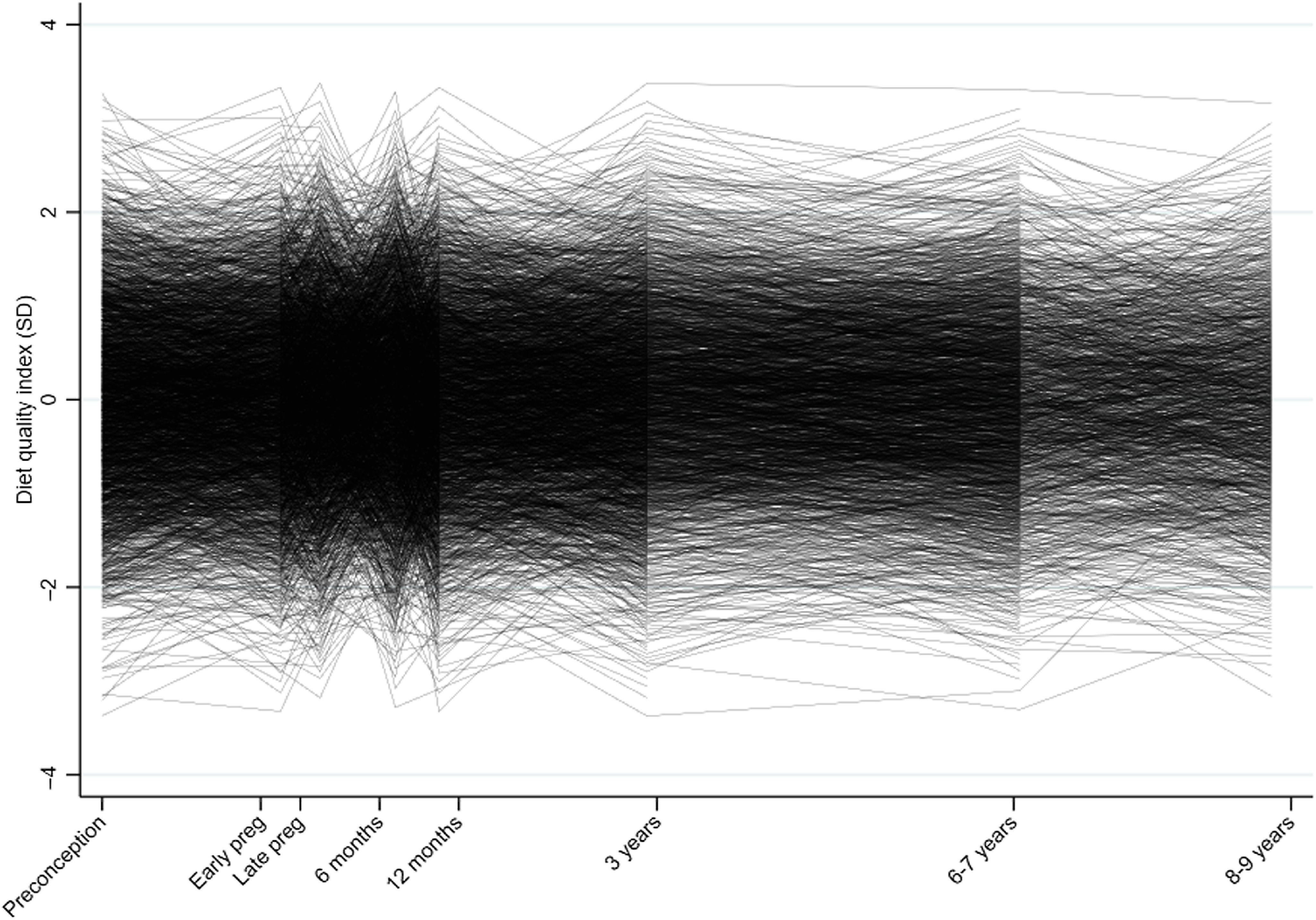
Individual trajectories of the diet quality index from preconception to 8–9 years of age.

**Fig. 3.**
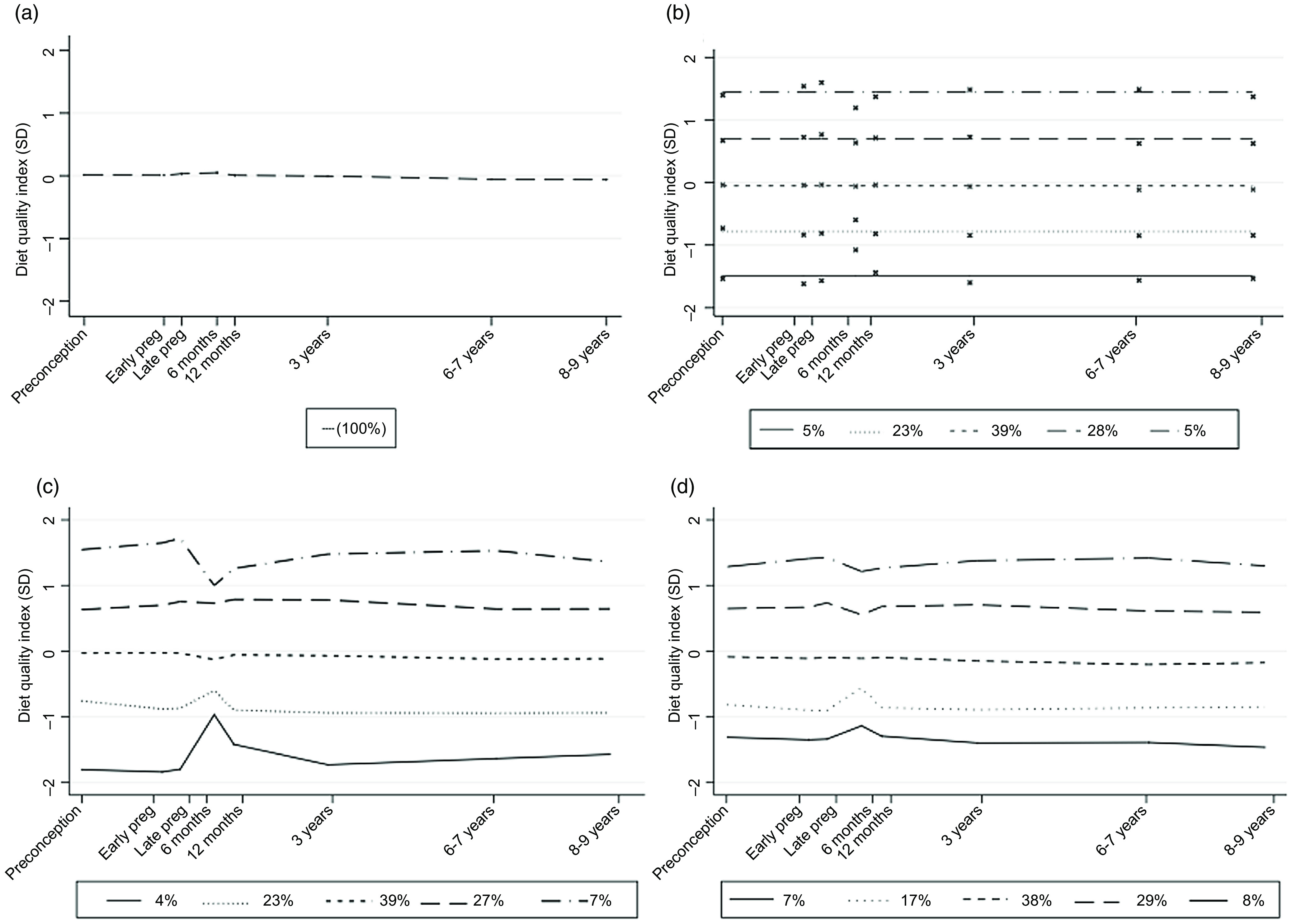
Latent class modelling representing (a) a growth curve model, (b) group-based trajectory model for five classes, (c) growth mixture model for five classes and (d) mean diet quality scores at each time point according to the group of the DQI across early life.

Figure 2 is a spaghetti plot that illustrates the individual diet quality trajectories. Pearson’s correlations coefficients between DQI at different time points ranged between 0·34 and 0·81 with higher correlations for ages/gestations closer in time. Supplementary Fig. 1 is a subsample of the SWS data (a random ten participants from each trajectory), and the figure illustrates the individual trajectories for these participants over time, categorised by class. Supplementary Table 1 details the number of participants with data at each time point; at least 80 % of participants had five or more data points. The overall pattern of missingness was defined as intermittent, as missing values are followed by observed data; we therefore assumed that any missing data were missing at random^(45)^. Figure 3(a) illustrates the latent class growth curve model (as described in step 1, part b).

### Group-based trajectory modelling

We compared GBTM from one to six classes and assessed each model using the model fit criteria (Table 2). Using the BIC, the preferred shape of the trajectories was found to be the intercept specification (a flat line). The one to four class models are shown in Supplementary Fig. 2. The five-class model was the best fit for the data (Table 2, Fig. 3(b)); although the six-class model had a lower BIC -21098 *v*. -21094), this model was rejected as only 1 % of the population were assigned to the highest class (Table 2). Further, the six-class model did not agree with the findings from the GMM. The individual trajectories for the five-class model by class are shown in Supplementary Fig. 3.

**Table 2.** Group-based trajectory modelling fit criteria for two to six classes

	BIC	AIC	Group membership	APPA	Entropy	OCC
1	-24719	-24713	(1) 100 %	–	–	–
2	-22116	-22104	(1) 51 %	(1) 0·94	0·8	18·8
			(2) 49 %	(2) 0·94		18·4
3	-21356	-21338	(1) 24 %	(1) 0·90	0·78	34·7
			(2) 47 %	(2) 0·89		10·1
			(3) 29 %	(3) 0·91		30
4	-21187	-21163	(1) 20 %	(1) 0·88	0·76	34·5
			(2) 41 %	(2) 0·86		10·2
			(3) 31 %	(3) 0·84		12·6
			(4) 8 %	(4) 0·84		71·7
5	-21098	-21068	(1) 5 %	(1) 0·82	0·74	85·2
			(2) 23 %	(2) 0·81		16·4
			(3) 39 %	(3) 0·82		8·7
			(4) 28 %	(4) 0·83		14·6
			(5) 5 %	(5) 0·84		85·2
6	-21094	-21058	(1) 1 %	(1) 0·81	0·72	324·7
			(2) 9 %	(2) 0·74		27·9
			(3) 20 %	(3) 0·72		10·8
			(4) 37 %	(4) 0·79		7·74
			(5) 27 %	(5) 0·83		15·1
			(6) 5 %	(6) 0·83		83·1

BIC, Bayesian information criteria; AIC, Akaike information criterion; APPA, average posterior probability of assignment; OCC, odds of correct classification.

### Growth mixture modelling

We compared GMM from one to six classes and assessed each model using the same criteria as the GBTM (Table 3). The one to four class models are shown in Supplementary Fig. 4. As the number of classes increased from three to five, we observed a deviation in the trajectories at the 6-month time point. As a sensitivity analysis, we restricted the five-class analysis to participants who had complete dietary data at the 6-month visit (*n* 1869) and observed that the deviation at the 6-month time point was still present (online Supplementary Fig. 5). We therefore hypothesise that the deviation at 6 months may be due to the lower dietary diversity as at this age an infant may still be on a solely breast/formula milk diet or on a limited diversity weaning diet. Similarly to GBTM, the five-class model was the best fit for the data (Fig. 3(c)). The individual trajectories by class are shown in Supplementary Fig. 6. Unlike GBTM, the growth mixture model for the six classes would not converge.

**Table 3.** Growth mixture model fit criteria for two to six classes

	BIC	AIC	Group membership	APPA	Entropy	OCC	Correlation with GBTM
1	49 441	49 426	(1) 100·0 %	–	–	–	–
2	44 228	44 142	(1) 50 %	(1) 0·95	0·81	29·6	0·9805
			(2) 50 %	(2) 0·94		29·8	
3	42 728	42 572	(1) 23 %	(1) 0·92	0·8	53·5	0·98
			(2) 49 %	(2) 0·90		16·1	
			(3) 28 %	(3) 0·92		43·8	
4	42 581	42 356	(1) 24 %	(1) 0·90	0·71	47·5	0·9026
			(2) 29 %	(2) 0·76		11·7	
			(3) 19 %	(3) 0·73		15·1	
			(4) 28 %	(4) 0·91		40·5	
5	42 294	41 999	(1) 4 %	(1) 0·84	0·76	150·5	0·98
			(2) 23 %	(2) 0·84		25	
			(3) 39 %	(3) 0·85		13·8	
			(4) 27 %	(4) 0·85		21·8	
			(5) 7 %	(5) 0·86		107·6	
**6**	**–**	**–**	**–**	**–**	**–**	**–**	**–**

BIC, Bayesian information criteria; AIC, Akaike information criterion; APPA, average posterior probability of assignment; OCC, odds of correct classification.

The six-class model would not converge.

We characterised these trajectories as stable with horizontal lines and defined them as poor (GMM = 4 % and GBTM = 5 %), poor-medium (23 %, 23 %), medium (39 %, 39 %), medium-better (27 %, 28 %) and best (7 %, 6 %) diet quality. Since the five patterns for each method can be considered ordered, for each model, we compared the correlation between the two methods (Table 3). There was a strong correlation between the five-class models for the GMM and the GBTM (Spearman’s correlation = 0·98). Finally, we compared the five-class models for GMM and GBTM to the method which has been used previously in the SWS cohort to describe longitudinal patterns of dietary intake (Fig. 3(d)). The cross-tabulation for the three methods is in Supplementary Table 2. There was a strong correlation between this method and both of the latent class methods (Spearman’s correlation = 0·90).

## Discussion

In this study, we compared two latent class modelling strategies for identifying dietary trajectories across early life. We have described approaches and the model assessment criteria in detail and found that both of these methods are suitable for identifying dietary trajectories. We have also demonstrated how to interpret these parameters when performing latent class modelling in Stata. There was strong agreement (model assessment and Spearman’s correlation) for both methods that the five-class model was most appropriate to describe diet quality across early life using data from 2963 participants of the SWS. These trajectories were stable from preconception to age 8–9 years and were defined as poor (about 5 %), poor-medium (about 23 %), medium (about 39 %), medium-better (about 28 %) and best (about 6 %). A deviation was observed at the 6-month time point, which we believe to be a result of the low diversity of an infant’s diet at this age.

To our knowledge, no previous study has applied latent class methodology to dietary pattern data collected preconceptionally to the age of 8–9 years. We have shown that both GBTM and GMM are suitable to model dietary trajectories. Although both methods have different approaches for modelling longitudinal trajectories, we observed a strong agreement for the optimal number of classes. However, both methods have their limitations. The process of fitting group-based trajectory models in Stata involves fitting several models for each class with varying specification (e.g. intercept/linear/quadratic). The outputs of these models are then compared to ascertain the correct specification. Although the command is quick to run in Stata, this approach is time-consuming and it is influenced by the available data and the specific research question being addressed. For example, for a five-group model in the SWS cohort, there were 1024 different possible trajectory shapes. In our study, rather than comparing 1024 different models, we used the BIC and the model output to determine the correct shapes for the trajectories. This approach still involved fitting several variations of the five-class model. When using GBTM, the final decision about the most appropriate model for the data is ultimately a somewhat subjective judgement by the researcher. Furthermore, GBTM assumes no inter-individual differences in change within class, so the covariance structure is zero, which implies that all individuals within a class are homogeneous^(31)^. In contrast, using the gllamm command to fit growth mixture models in Stata was computationally more intensive, although it does provide greater flexibility as it allows for varying covariance structure within a class^(18)^. This variation is achieved by allowing individuals within the same latent class to have a varying diet quality trajectory, providing more modelling flexibility^(46)^. Therefore, we suggest that GMM and GBTM could be used to complement each other when defining latent class dietary trajectories. In the first instance, researchers could use GBTM to model dietary trajectories, and the preferred model could be confirmed using GMM.

We also compared the outputs of our GBTM and GMM models with a previous analytical technique used to describe patterns in dietary intake in the SWS cohort^(19)^. The reason for providing a comparison to this previous SWS technique was to allow researchers to have alternative modelling options. Interestingly, for the five-class model, the results were similar (Spearman’s correlation 0·90). Although the previous technique would be unable to determine varying shapes in trajectories, this method may be a suitable starting point for longitudinal dietary analyses, and it may be particularly appropriate to use if there are only two or three waves of data as it is relatively easy to undertake in any statistical software.

### Implications for future research

In this paper, we have demonstrated how to model dietary trajectories across early life. These methods could be applied to longitudinal data across any lifecourse stages, and in doing so they provide researchers and public health professionals with the tools to explore relationships between diet quality trajectories and boarder social determinants of health, such as social, environmental and economic determinants of dietary intake as well as longer term health outcomes. We have illustrated the tracking of diet across early life; the potential cumulative effect of diet quality during this critical stage of the lifecourse builds on the evidence from previous studies that have reported tracking of dietary habits across childhood^(17,47,48)^ and reported associations between poor diet quality with adverse health outcomes^(49–51)^ including higher BMI, adiposity and cardiovascular outcomes. Our findings emphasise the importance of preconception diet quality, as we have shown that diet quality tracks from before the mother is pregnant, across pregnancy and into childhood. Given the implications of poor diet quality for long-term health, our findings suggest that the preconception period may be an important time to improve population health. This finding has the potential to provide a focus of public health strategies aiming to improve diet quality across early life. Our observation supports the recommendations outlined in the UK Preconception Partnership strategy^(52)^, which highlights the crucial role that maternal pre-pregnancy health, including unhealthy dietary habits, can have on future child health. The Partnership recommends improving population level health, irrespective of pregnancy planning, and at an individual level for those planning to become pregnant^(53,54)^. Although the preconception period is an important time to intervene, there are other opportunities to improve diet quality across early life. There is growing awareness of the relationship between the food environment and psychological factors on dietary choices^(55)^, including evidence that eating behaviours moderate the associations between risk factors in the first 1000 d and adiposity outcomes at the age of 6 years^(56)^.

### Strengths and limitations

This study has several strengths. Notably, the SWS is a large longitudinal mother–child cohort and the only population-based cohort in Europe to have collected data from the mother preconceptionally. Assessments of participants have been made at multiple time points across early life. Also, the dietary patterns, obtained by PCA, are able to provide a broader picture of an individual’s diet compared with single-nutrient analyses. We have also shown that there is a strong correlation between the GBTM, GMM and a more traditional method for trajectory modelling and highlighted how these three methods could be used to complement each other. However, there are some limitations. We completed all analyses in Stata; the Lo–Mendell–Rubin-adjusted likelihood ratio test and the parametric-bootstrapped likelihood ratio test can both be used to assess model fit (*P* < 0·05 indicates better fit) but are both unavailable in Stata^(44)^. They are however available in the software package Mplus. Along with Mplus, R and Latent Gold are also able to perform GMM and GBTM, all three of which are able to compute more fit statistics and they provide greater modelling flexibility compared with Stata. Although dietary patterns derived using PCA are a validated method for describing dietary intake^(57)^, these involve several arbitrary decisions including consolidation of food items into groups. FFQ are also known to be associated with recall bias from the child’s main caregiver^(58)^, but validation studies of those used against food diaries have shown that FFQ can be used to rank the nutrient intakes of individuals^(19–22,24)^. In the SWS, we used a variety of different FFQ for the mother and her offspring depending on their age. Therefore, we had to perform the latent class analysis using natural scores. However, if a future analysis used the same FFQ over time and the study population did not change, then applied scores could be used as these would have a constant scale to compare between time points, which would have some advantages.

### Conclusion

Due to the increasing availability of longitudinal data and the development of latent class methodology, nutritional scientists and public health professionals have more opportunities to explore the relationship between diet quality and long-term health outcomes. In this paper, we have shown how to apply two of these methods and how they compare with a more traditional statistical approach. Each approach has strengths and weaknesses; therefore, they could be used to complement each other when describing the relationships between diet quality exposure over a period and outcomes of interest to examine the influence of the broader factors influencing diet.
